# Books and Authors

**Published:** 1906-05

**Authors:** 


**Minor and Operative Surgery, including Bandaging.** By **Henry R. Wharton,** M.D., Professor of Clinical Surgery in the Woman's Medical College; Surgeon to the Presbyterian Hospital, Philadelphia,, etc. Sixth edition, enlarged and revised. In one 12mo. volume of 642 pages, with 532 illustrations. Lea Brothers & Co., Publishers, Philadelphia and New York, 1905. (Cloth. $3.00 net).

This book is an old friend now paying us its sixth visit, with an enlarged scope. The author has added operative surgery to its field, limiting the latter, however, to such procedures as can be practised advantageously on the dead subject. The sections or parts of the book are eight, which consider, in consecutive order, bandaging, minor surgery, asepsis and antisepsis, fractures, dislocations, operations, amputations, and excisions, resections, and special operations. As the work now stands it may be given first place among the manuals of minor surgery. It is superbly illustrated and is one of the most helpful of the smaller surgeries that ever has been published.

**Gallstones and Their Surgical Treatment.** By **B. G. A. Moynihan,** M.S. (London), F.R.C.S., Senior Assistant Surgeon to Leeds General Infirmary, Leeds, England. Second edition, revised and enlarged. Octavo of 458 pages, illustrated. Philadelphia and London: W. B. Saunders & Company, 1905. (Cloth, $5.00 net; Half Morocco, $6.00 net).

Mr. Moynihan is an acknowledged master of this subject and has presented, by and large, the most acceptable treatise on gallstone disease from the viewpoint of the surgeon that has been written up to this time. Indeed, it is now almost universally admitted that this disease belongs to the domain of surgery; certainly obstructive gallstone disease has no other place.

The first edition of this work was exhausted within eight months of its publication, which is a pretty good index of professional opinion as to the classification of the disease, whether medical or surgical. This edition is some seventy pages larger than the first and contains an additional chapter, dealing with congenital abnormalities of the gallbladder and bile ducts. The text has been carefully revised and well considered opinions have been given due weight, though, as we said in our notice the first edition, the great value of this work is its personality; it is essentially the work of the author himself, and it gives expression to his own experience. The illustrations, too, are chiefly original and we may add with truthfulness that they are most excellent.

---

**Differential Diagnosis and Treatment of Disease.** A textbook for Practitioners and Advanced Students, by **Augustus Caillé,** M.D., Professor of Diseases of Children, New York Post-Graduate Medical School and Hospital, etc. With 228 illustrations in the text. Octavo pp. 867. New York and London: D. Appleton and Company. 1906. (Price: Cloth, $6.00 net).

Another work on diagnosis pure and simple perhaps would not be needed at this time, but this one combines differential diagnosis and treatment in such a pleasing and instructive way, as to make it not only acceptable but actually useful if not necessitous. After describing the technic of diagnosis and laboratory aids to clinical diagnosis, Professor Caillé devotes a short chapter to general therapeutic management; then he assigns considerable space, about 140 pages, to a consideration of the diseases of children. This, in some respects, may be fairly considered the most interesting part of the treatise. It discusses with perspicacity the maladies of childhood and teaches clearly how to recognise many of the minor ailments that often perplex in the extreme. The instructions, too, relating to tracheotomy and intubation are excellent.

In the adult many diseases of difficult diagnosis are presented with a clearness that removes doubt or obscurity. The author is terse but generally makes the reader comprehend his meaning. It must be remembered that he is writing for practitioners and advanced students in particular, hence presupposes a preliminary knowledge of the topics he presents.

**Lectures on Auto-Intoxication in Disease,** or Self Poisoning of the Individual. By **Charles Bouchard,** Professor of Pathology and Therapeutics; Member of the Academy of Medicine and Physician to the Hospitals, Paris. Translated, with a Preface and New Chapters added by **Thomas Oliver,** M.A., M.D., F. R. C. P., Professor of Physiology, University of Durham; Physician to the Royal Infirmary, New Castle-upon-Tyne; Formerly Examiner in Medicine, Royal College of Physicians, London. Second revised edition. Crown octavo, 342 pages, extra cloth. F. A. Davis Company, Publishers, 1914-16 Cherry Street, Philadelphia. 1905. (Price, $2.00 net).

Professor Bouchard is a teacher and an author too well known on this side of the Atlantic to need an introduction at our hands or pen. As a pathologist he stands in the front rank, and as a clinician he is not behind his Parisian colleagues, many of whom are so celebrated in that direction.

Toxemia as a cause of a variety of diseases forms an important part of the study for the doctorate at the present time, and autointoxication, or, as would seem to us more appropriate, autotoxemia in an essential branch of that topic. While it is understood that all ptomaines are not poisons, it is important to learn which are toxic. The part played by microorganisms in the development of ptomaines is also of significant import in this investigation as to the causes of disease.

The student as well as the physician will find in these lectures great aid in solving many obscure problems connected with the contagium vivum,—the infective process. In particular will the presentation of the toxic effect of the excretions be found of great value to the clinician. It is a book that pathologists and other laboratory workers as well as clinicians should study with great care.

